# Effectiveness and Implementation Outcomes of an mHealth App Aimed at Promoting Physical Activity and Improving Psychological Distress in the Workplace Setting: Cluster-Level Nonrandomized Controlled Trial

**DOI:** 10.2196/70473

**Published:** 2025-05-06

**Authors:** Kazuhiro Watanabe, Mitsuhiro Sato, Shoichi Okusa, Akizumi Tsutsumi

**Affiliations:** 1 Department of Public Health, Kitasato University School of Medicine, 1-15-1 Kitazato, Minami-ku, Sagamihara, 252-0374, Japan, 81 427789352; 2 Human Resources Unit, Fujitsu General Limited, Kawasaki, Japan; 3 TOSHIBA Health Insurance Society, Kawasaki, Japan

**Keywords:** eHealth, behavioral change, mobile phone, smartphone, workplace, depression, anxiety

## Abstract

**Background:**

Encouraging physical activity improves mental health and is recommended in workplace mental health guidelines. Although mobile health (mHealth) interventions are promising for physical activity promotion, their impact on mental health outcomes is inconsistent. Furthermore, poor user retention rates of mHealth apps pose a major challenge.

**Objective:**

This study aimed to examine the effectiveness and implementation outcomes of the smartphone app ASHARE in Japanese workplace settings, leveraging a deep learning model to monitor depression and anxiety through physical activity.

**Methods:**

This hybrid effectiveness-implementation trial was a 3-month nonrandomized controlled trial conducted from October 2023 to September 2024. Work units and employees were recruited and allocated to the intervention or active control group based on preference. The intervention group installed the ASHARE app, whereas the control group participated in an existing multicomponent workplace program promoting physical activity. Changes in physical activity and psychological distress levels were compared between the groups. User retention rates, participation rates, acceptability, appropriateness, feasibility, satisfaction, and potential harm were also assessed.

**Results:**

A total of 84 employees from 7 work units participated (67 from 5 units in the intervention group and 17 from 2 units in the control group). In total, 78 employees completed the 3-month follow-up survey (follow-up rate: 93%). Both groups showed increased physical activity, and the intervention group showed reduced psychological distress; however, the differences between groups were not statistically significant (*P*=.20; *P*=.36). In a sensitivity analysis of protocol-compliant employees (n=21), psychological distress levels were significantly reduced in the intervention group compared with the control group (coefficient=−3.68, SE 1.65; *P*=.03). The app’s 3-month user retention rate was 20% (12/61), which was lower than the participation rate in each component of the control programs. Implementation outcomes evaluated by employees were less favorable in the intervention group than in the control group, whereas health promotion managers found them to be similar.

**Conclusions:**

The ASHARE app did not show superior effectiveness compared with an existing multicomponent workplace program for promoting physical activity. An implementation gap may exist between health promotion managers and employees, possibly contributing to the app’s low user retention rate. Future research should focus on examining the effectiveness of strategies to get engagement from managers and from segments of employees with favorable responses in the workplace at an early stage.

## Introduction

### Background

Mental health conditions among workers are widespread and adversely affect critical outcomes. In 2019, an estimated 301 million working-age adults experienced anxiety, while approximately 280 million had depression [1]. Furthermore, many workers report subclinical symptoms such as psychological distress [2-4]. These mental health concerns lead to increased absenteeism, deteriorating quality of life, and overall well-being [5]. The global economic burden of mental health conditions is estimated at approximately US $1 trillion annually, primarily due to productivity losses [1,6,7]. Given their range of severity, early interventions and preventive measures are essential for managing mental health in workplace environments.

Promoting physical activity effectively treats and prevents depression and anxiety and plays a critical role in preventing mental health conditions [8]. A previous study has reported that promoting physical activity improves outcomes such as job stress, absenteeism, job performance, and turnover among workers [9]. It is among the most evidence-based interventions for preventing major mental disorders in workers and is recommended in the World Health Organization’s mental health at work guidelines [1,10]. Due to the high prevalence of physical inactivity and mental health conditions among workers, encouraging physical activity has become a key strategy for improving workplace mental health.

Mobile health (mHealth) interventions delivered through smartphones and other devices effectively promote physical activity. These interventions typically use basic behavior change techniques, such as psychoeducation, goal setting, monitoring and providing feedback on physical activity, sharing and competing regarding activity levels among participants, or offering rewards [11]. mHealth interventions offer high accessibility and cost-effectiveness to diverse populations [12], and their effectiveness in promoting physical activity has been confirmed in various groups, including patients and the general adult population [13-15]. However, the quality of scientific evidence remains low, and the effects are inconsistent [16]. Furthermore, effectiveness is not always observed when depression, anxiety, or stress-related indicators are used as outcomes [17,18]. Although numerous mHealth interventions and apps aimed at promoting physical activity have been developed to date, studies in Asia or targeting healthy workers remain limited [13,19]. Furthermore, most of these interventions do not primarily focus on improving mental health, nor do they measure related outcomes [15].

The biggest challenge is poor implementation. For example, when programs are provided through smartphone apps, usage often declines due to poor design, obstacles, excessive notifications or ads, and insufficient efforts to prevent dropouts. In Japan, health care app retention rates are extremely low, ranging from 3.5% to 4% by the 30th day [20]. Low use rates also pose a major challenge in research [21]. Workers recognized that physical activity effectively improved mental health and expressed a desire for individualized health services. However, they highlighted the limited benefits of nonleisure physical activities and prioritized sleep and rest over physical activity [22]. Low organizational- and employer or manager-level engagement in programs are also important barriers to successful implementation in the Japanese workplace [23]. Therefore, while mHealth interventions may be scientifically effective, their impact is limited by poor user engagement. Given these challenges, further research is needed for workplace mHealth interventions to promote physical activity specifically designed to improve mental health outcomes, and tailored to the needs of the healthy working population to increase their long-term engagement.

### Objectives

To bridge these research gaps, we developed a native smartphone app called ASHARE to encourage physical activity and alleviate psychological distress in the working population. Previous findings on the implemented deep learning model and a feasibility study have been published elsewhere [24,25]. Briefly, the app incorporates basic behavior change techniques to encourage physical activity, including self-monitoring, feedback, and user-driven data sharing. In addition, it uses a deep learning model to enable passive monitoring of depression and anxiety based on physical activity patterns [24]. The beta version of the ASHARE app did not effectively promote physical activity or alleviate psychological distress overall but reduced the number of participants with severe psychological distress [25]. This app may contribute to providing evidence for mHealth research on improving mental health through physical activity among healthy workers, an area that has been scarcely examined. In addition, the deep learning model with individualized feedback has the potential to enhance individual worker engagement and to encourage employers or managers to implement the app who face constraints in human resources and prioritization for health promotion. This study aimed to evaluate the effectiveness and implementation outcomes of the revised ASHARE app [26] compared with an existing multicomponent workplace program as an active control. Based on the substantial evidence supporting the effectiveness of mHealth apps that use basic behavior change techniques to encourage physical activity, along with the additional feature of the ASHARE app for passive monitoring of depression and anxiety, we hypothesized that the increase in physical activity and improvement in psychological distress in the intervention group would be significantly greater than those in the active control group. In addition, we explored the retention rate, acceptability, appropriateness, feasibility, and satisfaction with the app compared with the existing workplace program to assess its implementation in workplace settings.

## Methods

### Study Design

A 3-month superiority nonrandomized controlled trial was conducted to investigate the effectiveness and implementation outcomes of the revised full version of the ASHARE app. We adopted a hybrid effectiveness-implementation design. From October 2023 to September 2024, the authors recruited work units and company employees through personal networks and connections. When companies agreed to participate in the trial, health promotion managers were selected as representatives to communicate with the research teams and select participating work units. Employees from the selected work units were invited to participate and asked to complete an internet-based baseline survey provided by the research team. In addition, the research team assigned work units to either the intervention or active control group based on the preferences expressed by health promotion managers. We adopted a nonrandomized design because randomization would not have aligned with the work units’ decision-making process. In addition, requiring randomization could have resulted in low participation rates, potentially compromising the successful execution of measuring implementation outcomes. The intervention group installed and used the ASHARE app for 3 months, while the control group participated in the existing multicomponent workplace program to promote physical activity during the same period [27]. Immediately after completing the programs, the research team sent the employees the internet-based follow-up survey to measure the effectiveness and implementation outcomes (acceptability, appropriateness, feasibility, and satisfaction) of each program. Health promotion managers also assessed the implementation outcomes of each program. As an incentive, health promotion managers and employees received an Amazon gift certificate worth 3000 yen (US $20.5) upon completion of the 3-month follow-up survey. This manuscript follows the guidelines of nonrandomized evaluations of behavioral and public health interventions (TREND [Transparent Reporting of Evaluations with Nonrandomized Designs] statement; Checklist 1) and Standards for Reporting Implementation Studies (StaRI) statement [28,29] (Checklist 2).

### Participants

This hybrid nonrandomized controlled trial included work units, health managers, and employees. No eligibility criteria were set for the work units. Eligible health promotion managers were employees in charge of human resources, labor affairs, general affairs, or health management, or employees in charge of promoting mental health in the workplace, according to the guidelines of the Japanese Ministry of Health, Labour and Welfare. Eligible employees within work units were aged 18 years or older, capable of completing questionnaires in Japanese, and owning a personal smartphone. Employees were excluded if they were absent during the enrollment period or had been absent within the previous 12 months.

### Interventions

#### Intervention Group

Employees in the work units assigned to the intervention group were instructed to install the ASHARE app on their smartphones and use it for 3 months. ASHARE is a native app compatible with Android (version 5.0 and later) and iOS (version 12.0 and later) devices. Following an initial feasibility trial [25], the fully developed iOS and Android app versions were made available through Google Play and the App Store, respectively [26]. The app uses basic behavior change techniques to encourage physical activity, including self-monitoring, feedback, and user-to-user data sharing. It integrates with Google Fit (Android) and Apple Health (iOS) to track the duration of physical activity. Each time the user launches the app, demographic and physical activity data from the previous day are sent to a cloud server. The model, using long short-term memory, calculates the predicted psychological distress for the present day, and predicted scores are displayed on the app. After the initial trial [25], several modifications were made to backend processing, feedback messages, the display of predicted depression and anxiety scores, graph tracking score changes, and feedback to the prediction algorithm. Furthermore, related content, YouTube channels, RSS feeds, a social networking account (X), and promotional videos were added. During the intervention period, employees were not forced to use the app, they could open it anytime. In terms of ideal app usage, the researchers anticipated that participants would spend approximately 5 min each day launching the app to review their physical activity patterns and the results of their predicted mental health scores while simultaneously aiming to increase their physical activity. To improve adherence to the program, researchers sent weekly email reminders to use the app. The app also sends users smartphone notifications with new information, and their predicted depression and anxiety scores were provided daily at 6 am. In addition, we monitored participants’ login frequency through the management dashboard during the study period once a week.

#### Control Group

Employees in the work units allocated to the control group were requested to participate in an existing multicomponent workplace intervention program featuring environmental changes for 3 months. The effectiveness of the program in promoting physical activity has been confirmed in Japanese workplace settings in a previous cluster randomized controlled trial [27]. The program comprises 13 elements across the different workplace components, including policymaking and declarations, posters detailing the program contents and physical activity recommendations, prompts for stair use near stairs and elevators, physical activity competitions between individuals within work units, and psychoeducation to increase self-regulation of physical activity. These elements, as evidenced by a literature review, build awareness and social norms around physical activity, enhance accessibility, and strengthen individual cognitive-behavioral skills [30-32]. Due to limited resources, only the elements considered relevant or feasible for each work unit were conducted after discussions with health promotion managers. To improve adherence to the program, researchers sent weekly email reminders to all participants in the work units. In addition, we monitored program participation through responses to the weekly survey, which tracked the number of steps for the physical activity competition.

### Outcomes

#### Physical Activity

The primary outcome measure was total physical activity, which was assessed using the Japanese version of the Global Physical Activity Questionnaire (GPAQ) [33]. This scale is widely used to evaluate physical activity levels in epidemiological studies and has demonstrated acceptable reliability and convergent validity across multiple countries, including Japan [34]. The intensity of physical activity was measured in metabolic equivalents (METs). As a continuous indicator, the total amount of physical activity per week (MET-hours/week) was calculated according to the GPAQ analysis guidelines [35]. Furthermore, physical activity levels were classified into 3 ordinal categories: low, moderate, and high.

#### Psychological Distress

The secondary outcome, self-reported psychological distress, was measured using the 6-item Kessler Psychological Distress Scale (K6) [36]. This scale was also used as the supervised indicator when the deep learning model was implemented in the ASHARE app [24]. The K6 includes 6 items that evaluate the frequency of symptoms related to depression and anxiety, rated on a 5-point Likert scale (0=none of the time to 4=all the time). The Japanese version of the K6 has been confirmed to be valid [37]. The total K6 score was calculated as a continuous variable. Levels of psychological distress were also categorized into 3 groups based on the proposed cut-off points: light level (<5), subthreshold level (≥5 and <13), and severe level (≥13) [37,38].

#### Implementation Outcomes

As an objective measurement of the program implementation, the user retention rate of the app (intervention) and the participation rate for each element of the program (control) were calculated. In the intervention group, app usage logs during the intervention period were retrieved from the app’s cloud server. The user retention rate was determined weekly; employees who launched the app at least once per week were classified as continuing users.

Participants in the control group were asked whether they had read the policy on physical activity promotion or had watched the psychoeducational video provided by the research team. Participation rates in the physical activity competition were calculated from questionnaire logs registering the amount of physical activity during the period.

For the subjective assessment of the program implementation by the employees, the Implementation Outcome Scale of Digital Mental Health (iOSDMH) was used [39]. This scale consists of 19 items assessing 5 domains: acceptability, appropriateness, feasibility, satisfaction, and potential harm. Each item is rated on a 4-point Likert scale (1=disagree to 4=agree). The scale’s reliability and validity have been verified in previous studies [39,40]. Subscale scores were calculated for each domain, and the overall score included the domains of acceptability, appropriateness, feasibility, and satisfaction. Furthermore, the iOSDMH for managers and policymakers was used to assess implementation outcomes by health promotion managers. This scale comprises 14 items covering the same domains as those for the user’s version: acceptability, appropriateness, feasibility, satisfaction, and potential harm. Unlike the users’ version, the version for managers and policymakers is rated on a 5-point Likert scale. The additional choice is “5=don’t know.” For score calculation, the average subscale score was calculated after excluding items rated as “don’t know.”

#### Demographic Variables

Age group (≤19, 20‐29, 30‐39, 40‐49, 50‐59, 60‐69, ≥70 years), gender (men, women, not specified, and other), employment status (full-time, contract, part-time, and dispatched), working shifts (day shift, rotation, and night shift), occupation (manager; professional, engineer, or academic; clerk; sales; service, transportation; construction; production; agriculture, forestry, or fishery; security; and other), and working hours per week (1‐34, 35‐40, 41‐50, 51‐60, 61‐65, 66‐70, ≥71) were recorded as demographic variables. These were used to calculate a propensity score to control baseline imbalances between the intervention and control groups.

### Sample Size Calculation

The required sample size was calculated at the individual (employee) level, although the work units were allocated to the 2 groups. The intraclass correlation coefficients for the primary and secondary outcome measures, physical activity, and psychological distress are known to be small in Japanese workplace settings; only 1% of variances are shared at the work-unit level [41,42]. The scientific evidence for using mHealth interventions to promote physical activity and reduce psychological distress is not well established, and the effect sizes are unavailable. In a previous meta-analysis, the standardized mean difference of mHealth interventions for increasing the number of steps and physical activity was estimated at 0.24 [43]. Based on this information, the required sample size was estimated as 274 employees per group (548 in total) using a significance level of .05 (2-sided) and statistical power (1-β) of 0.8 with G*Power (version 3.1.9.2, Heinrich Heine Universität, Düsseldorf) [44]. However, the required sample size could not be reached within the funded period, necessitating premature termination of recruitment and analysis.

### Statistical Analysis

At the individual level, the demographic characteristics of the employees at baseline were compared between the intervention and control groups using *t* tests or chi-square tests. To investigate the effectiveness of the ASHARE app on physical activity and psychological distress, generalized linear models were used to estimate the interaction effects of time (baseline and 3-month follow-up) by group (intervention and control). Based on the high follow-up rates in this study, we included data from employees who completed the 3-month follow-up survey in the analysis. Although both physical activity (MET-hours/week) and psychological distress can be treated as continuous variables, their distributions were skewed with high variances. Therefore, we treated them as both continuous and binary variables. For the binary measures, the levels of physical activity and psychological distress were dichotomized into low and moderate or high for physical activity, and light and subthreshold or severe for psychological distress. To ensure comparability between the intervention and control groups, a propensity score was calculated and included as a covariate in the model. The propensity for intervention group allocation was estimated using a logistic regression model. This model included age group, gender, employment status, occupation, working hours, total physical activity, and baseline psychological distress scores as predictive variables. Modeling was implemented using “MIXED” for continuous outcomes and “GENLINMIXED” (binomial, logit link) for binary outcomes using SPSS (version 29.0, IBM Corp). The remaining analyses were performed using the same software.

For the sensitivity analysis, we conducted a per-protocol analysis focusing on employees who continued to use the ASHARE app (intervention) and who participated in all the elements of the multicomponent workplace intervention program conducted in their work units (control). To evaluate the implementation outcomes of each program, the user retention rate for the ASHARE app (intervention) and the participation rate for each program element (control) were compared. In addition, the mean scores of the implementation outcomes (acceptability, appropriateness, feasibility, and satisfaction) assessed by the employees and health promotion managers were compared between the 2 groups. Furthermore, we compared the demographic characteristics, implementation outcome assessments, and qualitative evaluations between the app users who remained engaged and who dropped out.

### Ethical Considerations

The study protocol was registered in the University Hospital Medical Information Network (UMIN) clinical trials registry (UMIN-CTR, ID: UMIN000052374, registered October 10, 2023) and approved by the Kitasato University Medical Ethics Organization (C22-137). Informed consent was obtained from all participants before completing the baseline survey. The data were anonymized when analyzed. As an incentive, health promotion managers and employees received an Amazon gift certificate worth 3,000 yen (US $20.5) upon completion of the 3-month follow-up survey.

## Results

### Participant Characteristics

**Figure 1.**
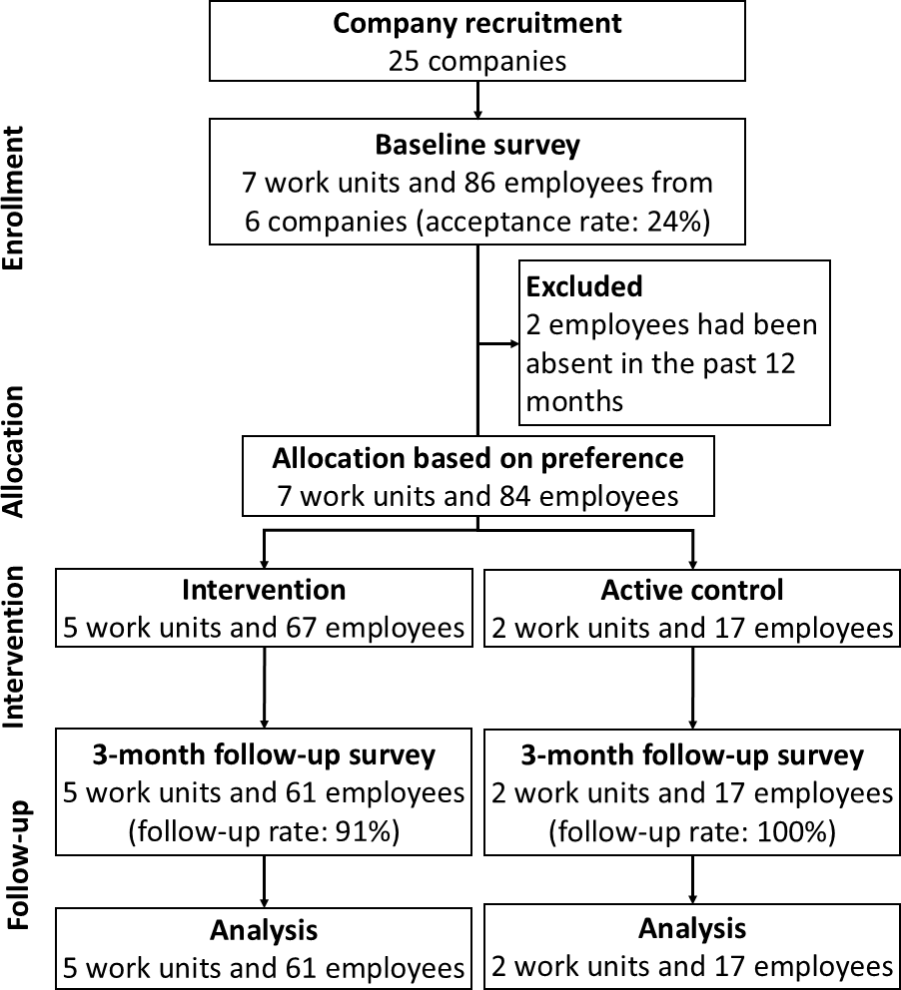
Participant flowchart.

Figure 1 illustrates the participant flow chart. A total of 7 work units and 86 employees from 6 companies participated (acceptance rate: 24%). After excluding 2 employees who had been absent due to illness in the previous 12 months, 5 work units and 67 employees preferred using the ASHARE app, while the other 2 work units and 17 employees preferred following the existing workplace program. The number of employees in the work units ranged from 7 to 22, with the mean number being 12 (SD 6.1). After completing the 3-month programs, 78 employees completed the follow-up survey (61 in the intervention group and 17 in the control group), and their data were included in the analysis (follow-up rate: 93%).

Table 1 presents the baseline demographic characteristics of the included employees. Most participants were full-time (65/78, 83%) and all were day shift (78/78, 100%) workers. Significant baseline imbalances in age group and employment status were found between the intervention and control group.

**Table 1.** Characteristics of the participants at baseline (N=78).

Characteristics	Total (N=78), n (%)	Intervention group (n=61), n (%)	Control group (n=17), n (%)	*P* value
Age group (years)				.03
20‐29	16 (21)	14 (23)	2 (12)	
30‐39	28 (36)	22 (36)	6 (35)	
40‐49	14 (18)	14 (23)	0 (0)	
50‐59	14 (18)	8 (13)	6 (35)	
≥60	6 (8)	3 (5)	3 (18)	
Gender				.93
Men	42 (54)	33 (54)	9 (53)	
Women	36 (46)	28 (46)	8 (47)	
Employment status				.02
Full-time	65 (83)	54 (89)	11 (65)	
Other	13 (17)	7 (12)	6 (35)	
Shift type				—^a^
Day shift	78 (100)	61 (100)	17 (100)	
Occupation				.14
Manager	10 (13)	7 (12)	3 (18)	
Professional, engineer, or academic	28 (36)	26 (43)	2 (12)	
Clerk	37 (47)	26 (43)	11 (65)	
Other	3 (4)	2 (3)	1 (6)	
Working hours per week (hours)				.53
1‐40	32 (41)	27 (44)	5 (29)	
41‐50	31 (40)	22 (36)	9 (53)	
51‐60	13 (17)	10 (16)	3 (18)	
≥61	2 (3)	2 (3)	0 (0)	

a Not applicable.

### Effect of an Intervention Program on Outcome Measures

Table 2 shows the physical activity and psychological distress levels of the 2 groups at baseline and the 3-month follow-up. Both groups, especially the group following the existing workplace program, showed increased physical activity after completing 3 months of the programs. Employees with moderate and high levels of physical activity saw an increase from 59% (36/61) to 66% (40/61) and from 53% (9/17) to 82% (14/17) at the 3-month follow-up, respectively; however, these changes were not statistically significant in the continuous (*P*=.98) or binary (*P*=.20) analyses.

The mean psychological distress score decreased from 3.87 to 3.75 in the ASHARE app group and increased from 4.18 to 4.59 in the active control group. In addition, the proportion of patients with subthreshold or severe levels of distress increased in the control group (from 29% [5/17] to 47% [8/17]), whereas it was maintained in the intervention group (from 31% [19/61] to 31% [19/61]). However, the group differences were not significant in the generalized linear model adjusted for the propensity score (*P*=.75; *P*=.36).

**Table 2.** Changes in physical activity and psychological distress levels after the 3-month interventions (N=78).

Outcomes	Intervention group (n=61)		Control group (n=17)		Effectiveness		
	Baseline	3-month follow-up	Baseline	3-month follow-up	Interaction effect of time by group	SE^a^	*P* value
Physical activity (GPAQ)^b^							
Total amount (MET-hours/week)^c^							
Mean (SD)	20.63 (18.5)	21.58 (20.3)	34.86 (73.2)	36.21 (48.2)	—^d^	—	—
Mean (SE)^e^	23.60 (5.2)	24.54 (4.1)	24.23 (11.5)	25.58 (9.9)	−0.4	12.84	.98
Level of physical activity^f^							
Low, n (%)	25 (41)	21 (34)	8 (47)	3 (18)	−1.16	0.91	.2
Moderate or high, n (%)	36 (59)	40 (66)	9 (53)	14 (82)	—	—	—
Psychological distress (K6)^g^							
Total score							
Mean (SD)	3.87 (3.3)	3.75 (4.3)	4.18 (5.8)	4.59 (4.9)	—	—	—
Mean (SE)^e^	4.13 (0.6)	4.01 (0.6)	3.25 (1.3)	3.67 (1.4)	−0.53	1.62	.75
Level of distress							
Light (≤4)	42 (69)	42 (69)	12 (71)	9 (53)	−0.78	0.84	.36
Subthreshold or severe (≥5)	19 (31)	19 (31)	5 (29)	8 (47)	—	—	—

a SE: standard error.

b GPAQ: Global Physical Activity Questionnaire.

c MET: metabolic equivalent.

d Not available.

e Estimated mean scores adjusted by propensity score.

f High: ≥ 3 days of vigorous-intensity activity of ≥1500 MET-min/week or ≥7 days of any combination of walking or moderate- to vigorous–intensity physical activity of 3000 MET-min/week; moderate: ≥3 days of vigorous-intensity activity of ≥20 min/day or ≥5 days of moderate-intensity activity or walking of ≥30 min/day or ≥5 days of any combination of walking or moderate- to vigorous–intensity physical activity of ≥600 MET-min/week; low: not meeting the criteria for high or moderate.

g K6: Kessler Psychological Distress Scale.

### Evaluation of User Retention Rate and Implementation Outcomes

Table 3 illustrates the user retention rate of the ASHARE app in the intervention group during the 3-month study period. The weekly user retention rate fell below 50% by the fourth week of the intervention and continued to decline steadily until the eighth week. In the last month, the retention rate stabilized at 20%‐30%. In the final week, the user retention rate was 20% (12/61), which was lower than the participation rate for each component of the control program: managers viewing the written policy (17/17, 100%), employees watching the psychoeducational YouTube (Google) video (9/17, 53%), and participation in the final week’s physical activity competition (14/17, 82%). In the control group, the percentage of employees who participated in all the elements conducted in the work units was 53% (9/17). When we compared app users in the intervention group, those who remained engaged were more likely to be in their 50s and employed as professionals, engineers, or academics. In contrast, those who dropped out were more likely to be over 60 and employed as clerks (Table S1 in Multimedia Appendix 1).

Table 4 presents the mean scores for acceptability, appropriateness, feasibility, satisfaction, and harm in the 2 groups, as measured using the iOSDMH. The implementation outcomes, as evaluated by the employees, were less favorable in the intervention group than in the control group, except for harm. Conversely, the implementation scores, as evaluated by health promotion managers, show little difference between the 2 groups. When we compared app users in the intervention group, no significant differences were observed between those who remained engaged and those who dropped out (Table S2 in Multimedia Appendix 2). However, the retention group rated the app’s feasibility slightly more favorably. Qualitative feedback from the dropout group indicated several concerns, including slow app launch times, a lack of specificity in the provided advice, and a desire for enhanced gamification features.

**Table 3.** User retention rate of the ASHARE app over 3 months during the intervention period (n=61).

Week	Retention users, n (%)
1	46 (75)
2	42 (69)
3	33 (54)
4	25 (41)
5	20 (33)
6	18 (30)
7	16 (26)
8	14 (23)
9	16 (26)
10	14 (23)
11	12 (20)
12	11 (18)
13	12 (20)

**Table 4.** Assessment of the implementation outcomes of the interventions.

Outcomes	Intervention group, mean (SD)	Control group, mean (SD)	*P* value for *t* test	Beta version of ASHARE^a^, mean (SD)
Employees (n=78), n	61	17	N/A^b^	23
Overall score (14 items; range 14‐56)	36.21 (7.2)	48.00 (5.4)	<.001	36.65 (5.1)
Acceptability (3 items; range 3‐12)	7.05 (1.9)	9.24 (1.5)	<.001	6.87 (1.5)
Appropriateness (4 items; range 4‐16)	9.87 (2.4)	13.65 (2)	<.001	9.96 (2.3)
Feasibility (6 items; range 6‐24)	17.00 (3.5)	21.59 (2.3)	<.001	17.74 (2.8)
Satisfaction (1 item; range 1‐4)	2.30 (0.8)	3.53 (0.5)	<.001	2.09 (0.7)
Harm (5 items; range 5‐20)	7.10 (2.8)	6.18 (1.5)	.19	6.43 (1.6)
Health promotion managers (n=7)	5	2	N/A	—^c^
Acceptability (4 items; range 1‐4)	3.3 (0.5)	3.63 (0.5)	.5	—
Appropriateness (4 items; range 1‐4)	3.35 (0.2)	3.42 (0.1)	.72	—
Feasibility (4 items; range 1‐4)	3.8 (0.1)	3.75 (0.4)	.76	—
Satisfaction (1 item; range 1‐4)	2.8 (0.8)	3.5 (0.7)	.35	—
Harm (1 item; range 1‐4)	1.2 (0.4)	1 (0)	.58	—

a Scores were obtained in our previous feasibility trial [25].

b Not applicable.

c Not available.

### Per-Protocol Analysis Among Program Completers

For the sensitivity analysis, the program effectiveness was investigated among 21 completers (12 in the intervention group and 9 in the control group). Table 5 presents the results of the generalized linear modeling. The analyses revealed more favorable changes in physical activity and psychological distress levels in the intervention group compared with the control group. Although these differences were not significant in terms of physical activity, psychological distress significantly decreased. The proportion of the subthreshold or severe distress levels in the intervention group was significantly reduced compared with the control group (coefficient=−3.68, SE 1.65; *P*=.03).

**Table 5.** Sensitivity analysis: changes in physical activity and psychological distress among program completers (n=21).

Outcomes	Intervention group (n=12)		Control group (n=9)		Effectiveness		
	Baseline	3-month follow-up	Baseline	3-month follow-up	Interaction effect of time by group	SE^a^	*P* value
Physical activity (GPAQ)^b^							
Total amount (MET-h/week)^c^							
Mean (SD)	28.31 (25.5)	28.92 (25.1)	49.7 (99)	16.1 (9.9)	—^d^	—	—
Mean (SE)^e^	26.9 (20)	27.51 (7.7)	51.58 (23.4)	17.97 (9.4)	34.22	31.1	.28
Level of physical activity^f^							
Low, n (%)	4 (33)	3 (25)	2 (22)	2 (22)	0.41	1.54	.80
Moderate or high, n (%)	8 (67)	9 (75)	7 (78)	7 (78)	—	—	—
Psychological distress (K6)^g^							
Total score							
Mean (SD)	5.58 (4.1)	3.17 (3.3)	2.44 (1.9)	4.89 (4.5)	—	—	—
Mean (SE)^e^	6.47 (1.2)	4.06 (1.3)	1.26 (1.4)	3.70 (1.5)	–4.86	2.23	.04
Level of distress							
Light (≤4)	6 (50)	9 (75)	8 (89)	4 (44)	–3.68	1.65	.03
Subthreshold or severe (≥5)	6 (50)	3 (25)	1 (11)	5 (56)	—	—	—

a SE: standard error.

b GPAQ: Global Physical Activity Questionnaire.

c MET: metabolic equivalent.

d Not available.

e Estimated mean scores adjusted by propensity score.

f High: ≥ 3 days of vigorous-intensity activity of ≥1500 MET-min/week or ≥7 days of any combination of walking or moderate- to vigorous–intensity physical activity of 3000 MET-min/week; moderate: ≥3 days of vigorous-intensity activity of ≥20 min/day or ≥5 days of moderate-intensity activity or walking of ≥30 min/day or ≥5 days of any combination of walking or moderate- to vigorous–intensity physical activity of ≥600 MET-min/week; low: not meeting the criteria for high or moderate.

g K6: Kessler Psychological Distress Scale.

## Discussion

### Principal Findings

Although the per-protocol sensitivity analysis revealed a significant improvement in psychological distress, the primary results did not support the hypothesis that the ASHARE app was more effective than the existing workplace program. Regarding implementation, the user retention rate and employee assessment outcomes were lower than those of active controls across the various measured indicators. This highlights that the app still has several shortcomings in its design and functionalities, including slow launch times, unoptimized content, and a lack of integrated gamification elements. No statistically significant differences between the app and the existing workplace program were observed in the primary and secondary outcomes. Active control, an existing workplace intervention program with environmental changes, successfully increased employee’s physical activity levels. The developed app may not be more effective than traditional, face-to-face, multicomponent programs. The lack of a superior effect on increasing physical activity may be attributed to the overlap in behavior change techniques used in both programs. The psychoeducation and competitions in the control program were also designed to encourage self-monitoring and data sharing. Nevertheless, the changes in psychological distress, while not statistically significant, differed between the groups. This may be due to the aim of the app, which encourages the monitoring of depression and anxiety while highlighting the association between physical activity and mental health through a deep learning model. Although this study did not provide sufficient evidence, it may suggest the potential of the deep learning model for outcome-specific effects in promoting physical activity.

The biggest challenge in implementing mHealth interventions in workplace settings was not addressed in this study. The user retention rate of the ASHARE app, 20% (12/61)at the 3-month follow-up, was not very low; it was higher than those of health care apps on the market (3.5%‐4%) and the 4-week follow-up rate in a previous intervention study (35.7%) [20,21]. Weekly reminders sent by the researchers in this study may have contributed to maintaining the user retention rate. However, this was insufficient to achieve favorable effects in the work units. Furthermore, compared with the active control group, employees rated the app more poorly across the dimensions of acceptability, appropriateness, feasibility, and satisfaction. Despite prerelease modifications, the ASHARE app’s overall implementation score (36.21, SD 7.2) was similar to its beta version (36.65, SD 5.1) [25]. Conversely, assessments of the control program were mostly favorable. Discrepancies may have occurred between research and practical settings in the control group. Existing workplace intervention programs require substantial coordination from providers, including drafting written policies, distributing promotional materials (eg, flyers and posters), organizing psychoeducation sessions, and overseeing competitive activities. In this study, researchers managed these responsibilities instead of health promotion managers, effectively exempting the latter from typical program-related efforts. Under these conditions, the acceptability and feasibility of traditional face-to-face multicomponent programs in practical settings may be lower than those in this present study.

Some valuable insights were obtained from the comparison between the retention and dropout groups among app users, as well as from the qualitative feedback provided by those who dropped out. The retention group primarily consisted of younger, professional workers who exhibited higher levels of distress at baseline. These segments of workers could serve as the initial target for promoting the implementation of the app in the workplace. In contrast, older workers may require more intensive support to encourage app engagement. Improvements to the app’s design are also necessary: the ASHARE app exchanges data with the server upon launch to predict mental health scores, which may contribute to slower startup times. In addition, since the program is written in Python, an interpreted language, its processing speed may be slower compared with compiled languages such as C++. Optimizing the program or converting it to a compiled language could help address user dissatisfaction related to slow launch times. Following a user-centered approach, enhancing the specificity and diversity of feedback messages, as well as incorporating gamification elements, such as badges, points, narratives, and characters could further improve user engagement [45]. An alternative explanation is that work-unit level participation could make them feel compelled, leading to fatigue in using the app.

A potential area for improving the app’s effectiveness is addressing the evaluation discrepancy between app users (employees) and health promotion managers. While managers did not rate the app poorly regarding implementation outcomes, employees suggested room for improvement. This discrepancy indicates an implementation gap between managers and employees, potentially affecting the app’s overall effectiveness. This suggestion is inconsistent with the previous findings from the qualitative study in Japan [23]. Future implementations may benefit from additional strategies, such as informational sessions on the app’s objectives, benefits, and usage to enhance employees’ understanding and encourage greater commitment to the program.

The per-protocol analysis indicated a significant reduction in psychological distress in the intervention group. Since the results were based on a post-hoc analysis and the baseline imbalance was more noticeable than in the overall sample, they must be carefully interpreted. This reduction is merely a regression to the mean. Despite these limitations, the app’s efficacy in reducing psychological distress may be favorable if employees continuously use the app for 3 months. Furthermore, based on the characteristics of the program completers, the app may be particularly efficacious in users with lower activity and higher psychological distress. This aligns with findings from our feasibility trial, where the number of participants experiencing severe psychological distress decreased after using the app [25]. Future implementations may need to identify appropriate workplace intervention targets.

This study had a few limitations. First, we could not reach the required sample size within the funded period, leading to the termination of recruitment at our convenience. This limitation led to low statistical power in detecting the significant improvement in outcomes and disturbed the hypothesis-testing framework. In addition, the imbalance in allocation between the intervention and control groups may increase the risk of both type I and type II errors. The nonrandomized design has inherent biases that could not be adjusted even if propensity scores were used in statistical modeling. Other unmeasured confounders may include motivation for health promotion, job stress, and medical history. We adopted a nonrandomized design because randomization would not have agreed with the work units’ decision-making process. Therefore, additional efforts are needed to ensure the success of cluster-randomized controlled trials. The outcome measures and implementation outcomes rely heavily on self-reported data, which are prone to recall bias and social desirability bias. Even if the program had been offered to the control group, given the study setting designed to investigate the effects of the newly developed mHealth app, participants in the intervention group would still be more likely to report favorable changes. Since most participants were full-time workers on day shifts, the generalizability of the findings is limited. In addition, compared with the overall population of Japanese workers, the participants in this study included a higher proportion of professionals. Other working populations, especially night-shift workers, were not covered in this study.

### Conclusions

The ASHARE app did not show superior effectiveness compared with the existing workplace program. An implementation gap may exist between health promotion managers and employees, possibly contributing to the app’s low user retention rate. Future research should focus on improving the app design identified in this study, as well as examining the effectiveness of strategies to get engagement from managers and from segments of employees with favorable responses in the workplace at an early stage. Also for apps already implemented in society, strategies may be needed to bridge the engagement gap between decision-making managers and employees. Furthermore, efforts to ensure the success of cluster-randomized controlled trials will be necessary to generate more robust and reliable findings.

## Supplementary material

10.2196/70473 Multimedia Appendix 1 Characteristics of the app users who retained and dropped out (n=61).

10.2196/70473 Multimedia Appendix 2 Assessment of the implementation outcomes among the app users who retained and dropped out (n=61).

10.2196/70473 Checklist 1 A completed TREND checklist.

10.2196/70473 Checklist 2 A completed StaRI checklist.

## Abbreviations

GPAQ: Global Physical Activity Questionnaire
iOSDMH: Implementation Outcome Scale of Digital Mental Health
K6: 6-item Kessler Psychological Distress Scale
MET: metabolic equivalent
StaRI: Standards for Reporting Implementation Studies
TREND: Transparent Reporting of Evaluations with Nonrandomized Designs
UMIN: University Hospital Medical Information Network
