# Supplementary material for: Effectiveness and Implementation Outcomes of an mHealth App Aimed at Promoting Physical Activity and Improving Psychological Distress in the Workplace Setting: Cluster-Level Nonrandomized Controlled Trial
Source: JMIR Mhealth Uhealth. 2025 May 6;13:e70473. doi: 10.2196/70473 (PMC12071197; doi:10.2196/70473)
Supplement: Multimedia Appendix 1 [file mhealth-v13-e70473-s001.pdf]

Supplement Table 1. Characteristics of the app users who retained and dropped out(n = 61)

	Intervention group (n = 61) n (%)	Retention (n = 12) n (%)	Dropout (n = 49) n (%)	*P*-value
Age group (years)				
20–29	14 (23)	2 (17)	12 (25)	
30–39	22 (36)	3 (25)	19 (39)	
40–49	14 (23)	2 (17)	12 (25)	.03
50–59	8 (13)	5 (42)	3 (6)	
≥60	3 (5)	0 (0)	3 (6)	
Gender				
Male	33 (54)	6 (50)	27 (55)	.75
Female	28 (46)	6 (50)	22 (45)	
Employment status				
Full-time	54 (89)	10 (83)	44 (90)	.53
Other	7 (12)	2 (17)	5 (10)	
Shift type				
Day shift	61 (100)	12 (100)	49 (100)	–
Occupation				
Manager	7 (12)	2 (17)	5 (10)	
Professional/ Engineer/Academic	26 (43)	7 (58)	19 (39)	.42
Clerk	26 (43)	3 (25)	23 (47)	
Other	2 (3)	0 (0)	2 (4)	
Working hours per week (hours)				
1–40	27 (44)	5 (42)	22 (45)	
41–50	22 (36)	4 (33)	18 (37)	.75
51–60	10 (16)	2 (17)	8 (16)	
≥61	2 (3)	1 (8)	1 (2)	
