# Supplementary material for: Effectiveness and Implementation Outcomes of an mHealth App Aimed at Promoting Physical Activity and Improving Psychological Distress in the Workplace Setting: Cluster-Level Nonrandomized Controlled Trial
Source: JMIR Mhealth Uhealth. 2025 May 6;13:e70473. doi: 10.2196/70473 (PMC12071197; doi:10.2196/70473)
Supplement: Multimedia Appendix 2 [file mhealth-v13-e70473-s002.pdf]

Supplement Table 2. Assessment of the implementation outcomes among the app users who retained and dropped out(n = 61)

	Intervention group (n = 61) Mean (SD)	Retention (n = 12) Mean (SD)	Dropout (n = 49) Mean (SD)	*P*-value for *t*-test
Overall score (14 items; 14–56)	36.21 (7.2)	35.58 (6.6)	36.37 (7.3)	.37
Acceptability (3 items; 3–12)	7.05 (1.9)	6.83 (2.3)	7.10 (1.8)	.67
Appropriateness (4 items; 4–16)	9.87 (2.4)	8.92 (2.8)	10.1 (2.3)	.13
Feasibility (6 items; 6–24)	17.00 (3.5)	17.67 (1.6)	16.84 (3.9)	.47
Satisfaction (1 item; 1–4)	2.30 (0.8)	2.17 (0.9)	2.33 (0.8)	.56
Harm (5 items; 5–20)	7.10 (2.8)	7.25 (1.9)	7.06 (3.0)	.84
